# Supplementary material for: Leucine Supplementation Prevents the Development of Skeletal Muscle Dysfunction in a Rat Model of HFpEF
Source: Cells. 2024 Mar 13;13(6):502. doi: 10.3390/cells13060502 (PMC10969777; doi:10.3390/cells13060502)

WB evaluation of porin

Membrane was treated as followed
- Blocking 5% milk in TTBS
- anti-porin (1:1000, 2h, RT)
- Anti-rabbit POD (1:10´000, 1h, RT)
- Developed
- Anti-GAPDH (1:5000, 1h, RT)
- Anti-mouse POD (1:5000, 1h, RT)
- developed

Ob = HFpEF untreated
Pr = HFpEF primary prevention
Se = HFpEF secondary prevention

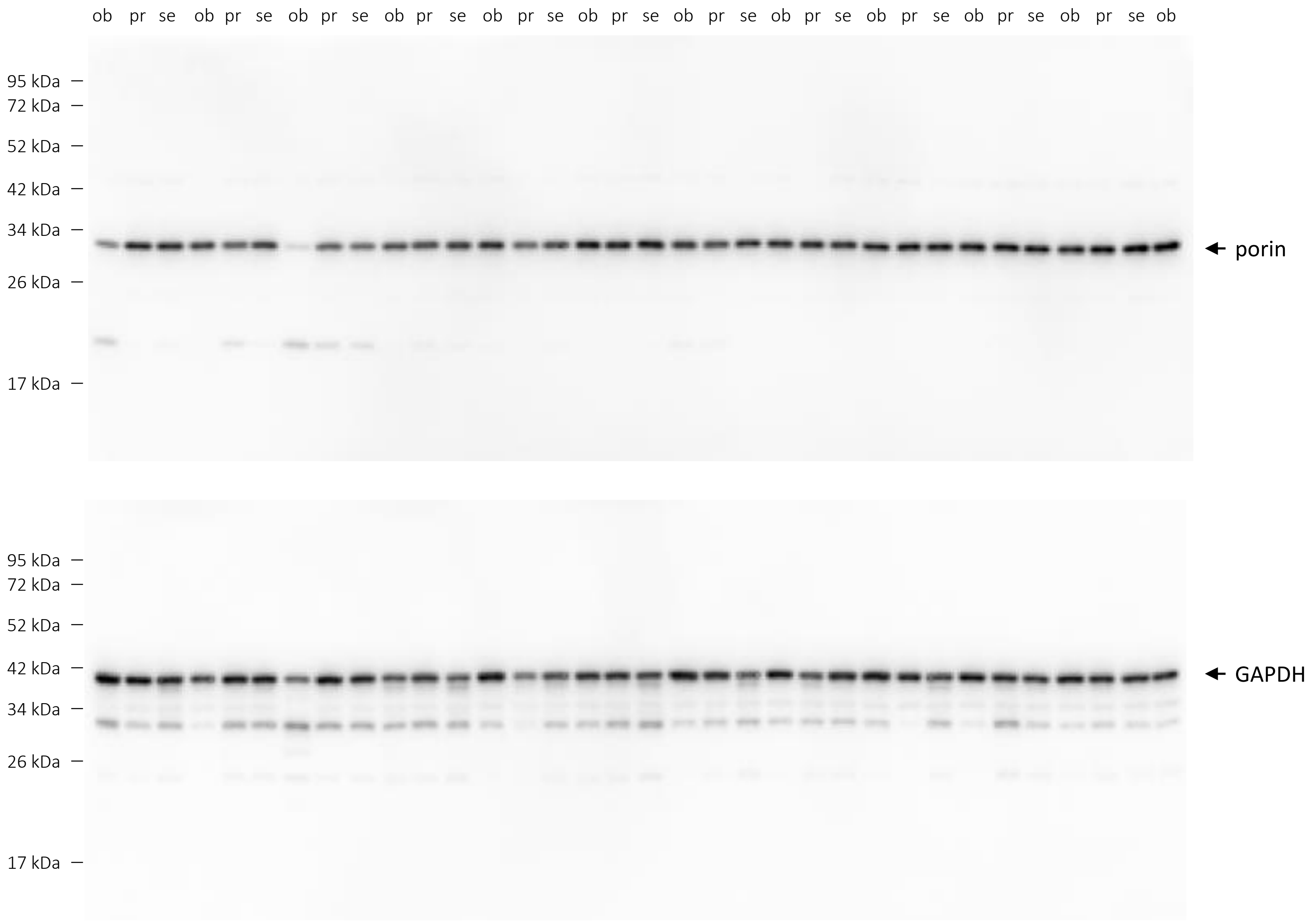

WB evaluation of mitochondrial respiratory chain complex proteins

Membrane was treated as followed

- Blocking 5% milk in TTBS
- anti-total OXPHOS (1:250, O/N, 4°C)
- Anti-mouse POD (1:5000, 1h, RT)
- Developed
- Anti-GAPDH (1:5000, 1h, RT)
- Anti-mouse POD (1:5000, 1h, RT)
- developed

Ob = HFpEF untreated
Pr = HFpEF primary prevention
Se = HFpEF secondary prevention

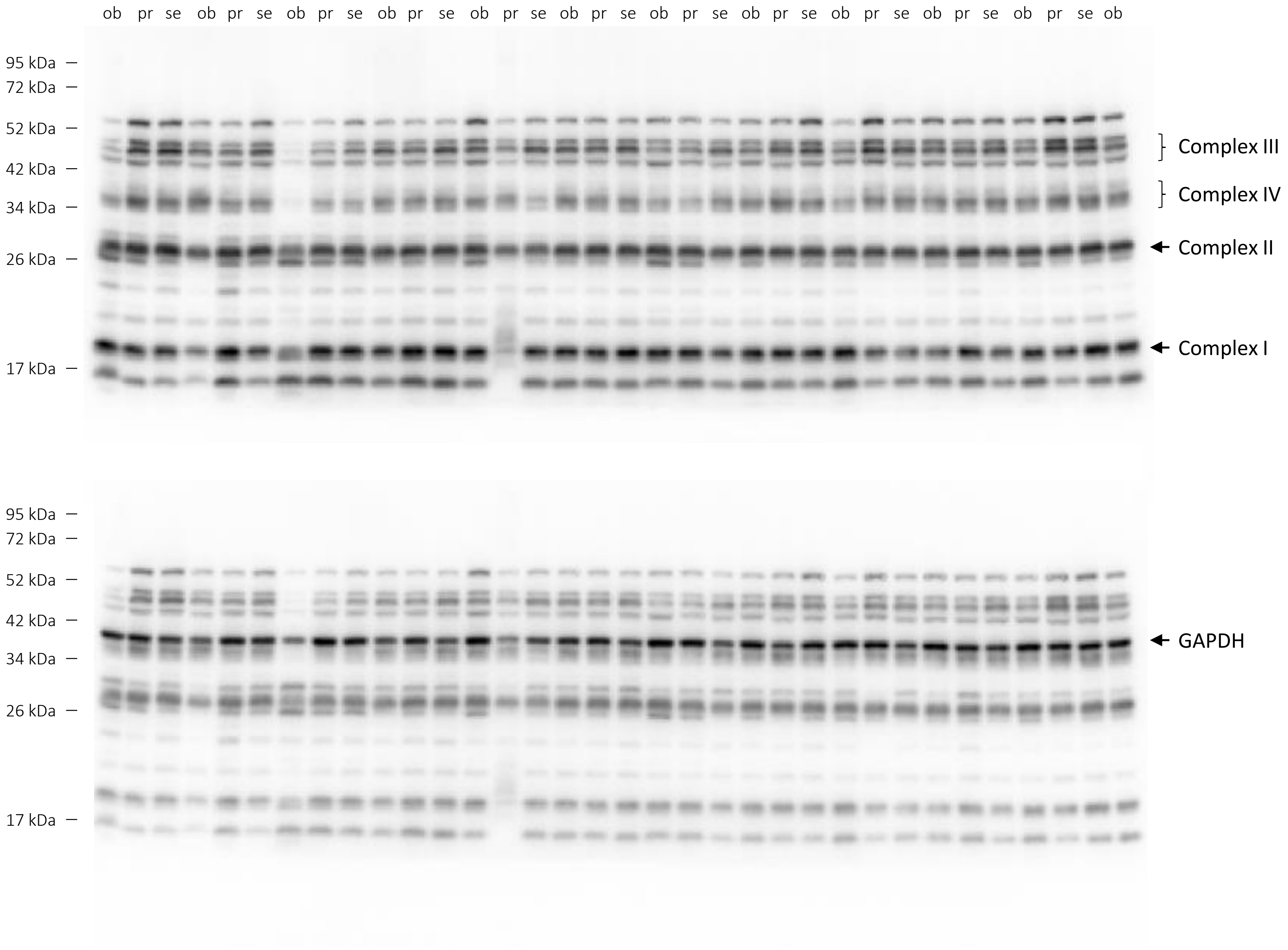

WB evaluation of FIS1

Membrane was treated as followed
- Blocking 5% milk in TTBS
- anti-FIS1 (1:1000, O/N, 4°C)
- Anti-rabbit POD (1:10´000, 1h, RT)
- Developed
- Anti-GAPDH (1:5000, 1h, RT)
- Anti-mouse POD (1:5000, 1h, RT)
- developed

Ob = HFpEF untreated
Pr = HFpEF primary prevention
Se = HFpEF secondary prevention

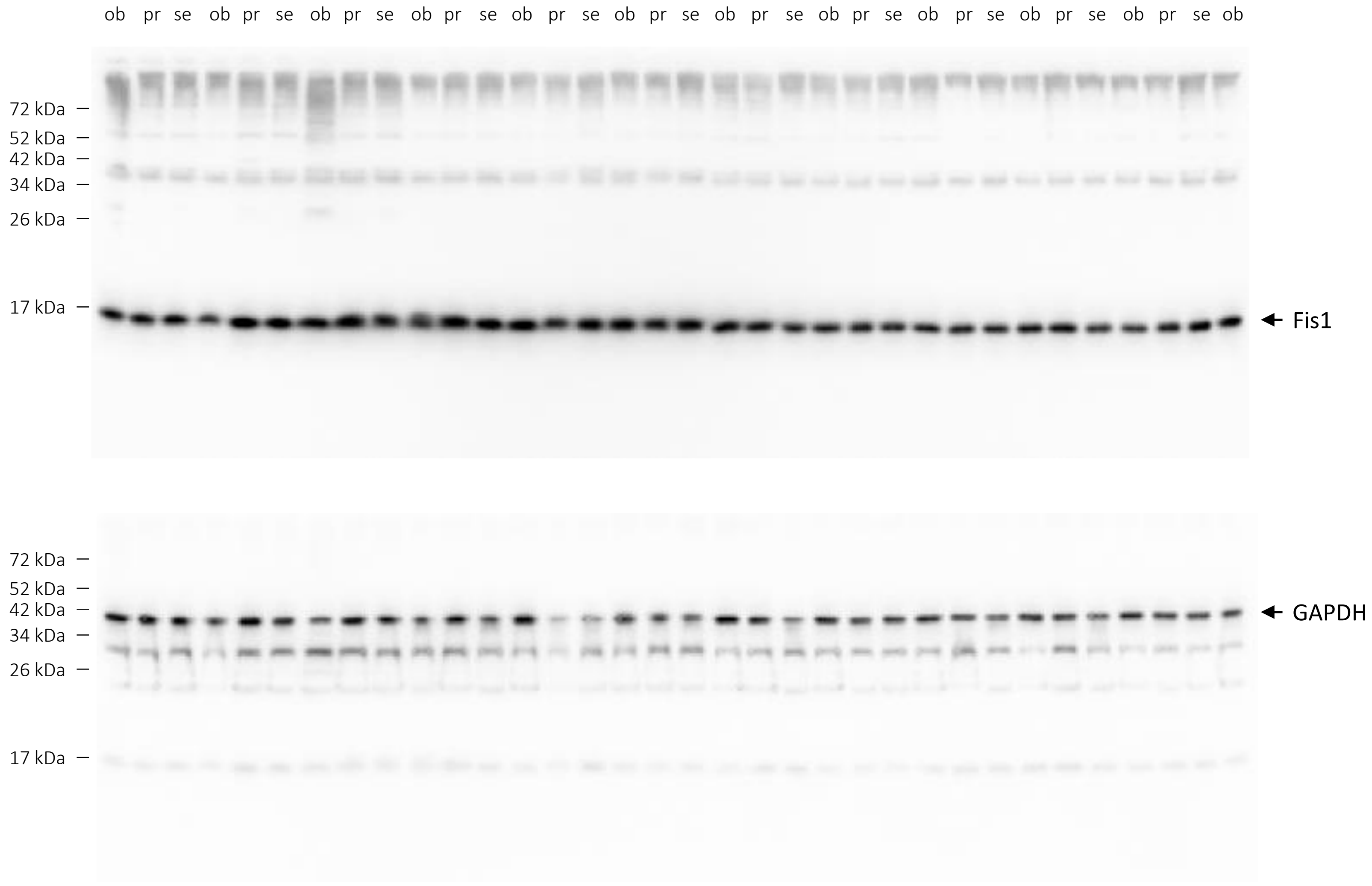

WB evaluation of MFN2

Membrane was treated as followed
- Blocking 5% milk in TTBS
- anti-MFN2 (1:1000, O/N, 4°C)
- Anti-rabbit POD (1:10´000, 1h, RT)
- Developed
- Anti-GAPDH (1:5000, 1h, RT)
- Anti-mouse POD (1:5000, 1h, RT)
- developed

Ob = HFpEF untreated
Pr = HFpEF primary prevention
Se = HFpEF secondary prevention

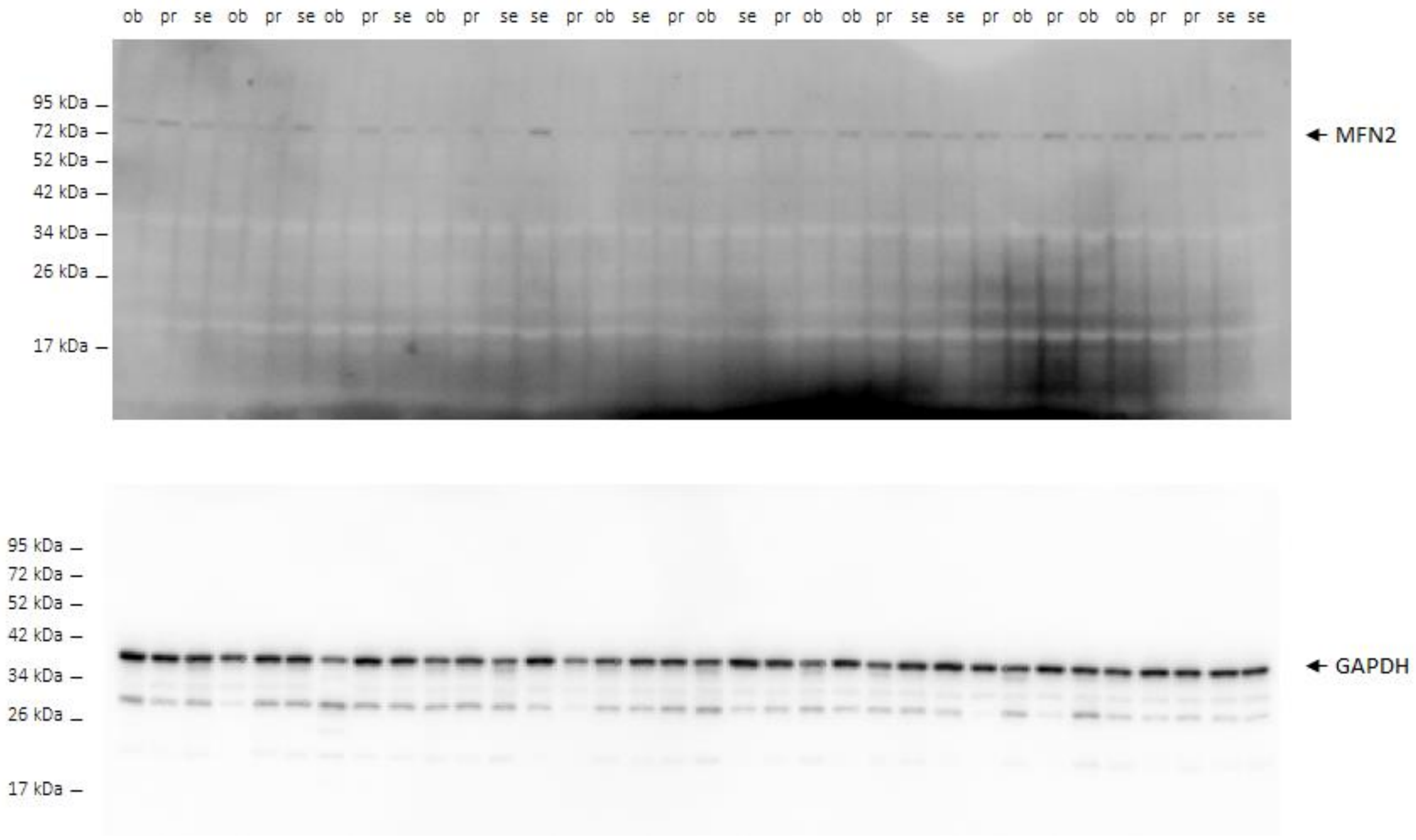

WB evaluation of mitofilin and mi-CK

Membrane was treated as followed

- Blocking 5% milk in TTBS
- anti-mitofilin (1:200, O/N, 4°C)
- Anti-mouse POD (1:5000, 1h, RT)
- Developed
- Anti-mi-CK (1:2000, 2h, RT)
- Anti-rabbit POD (1:10´000, 1h, RT)
- Developed
- Anti-GAPDH (1:5000, 1h, RT)
- Anti-mouse POD (1:5000, 1h, RT)
- Developed

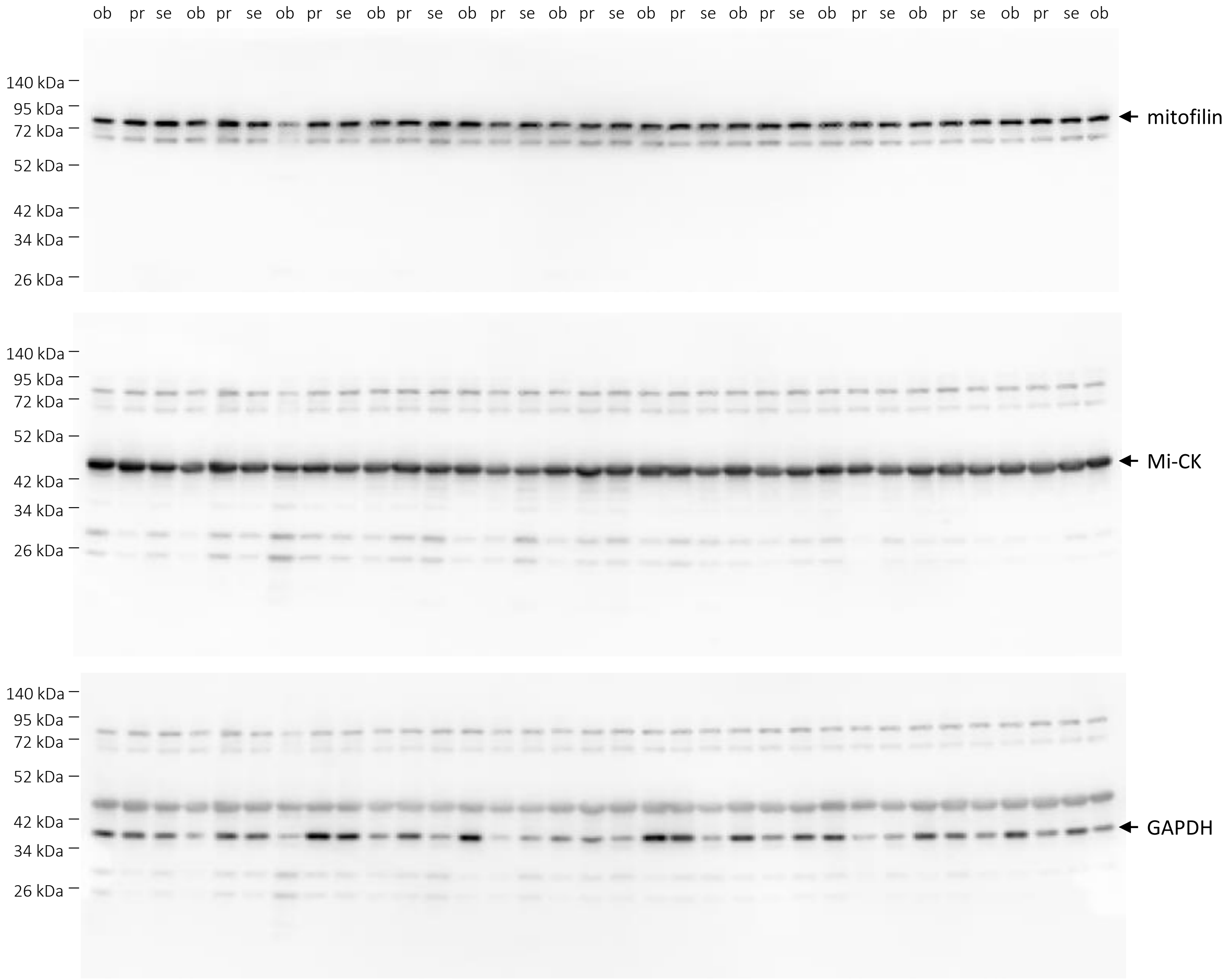

Ob = HFpEF untreated
Pr = HFpEF primary prevention
Se = HFpEF secondary prevention

WB evaluation of UCP3

Membrane was treated as followed

- Blocking 5% milk in TTBS
- anti-UCP3 (1:1000, O/N, 4°C)
- Anti-rabbit POD (1:10´000, 1h, RT)
- Developed
- Anti-GAPDH (1:5000, 1h, RT)
- Anti-mouse POD (1:5000, 1h, RT)
- developed

Ob = HFpEF untreated
Pr = HFpEF primary prevention
Se = HFpEF secondary prevention

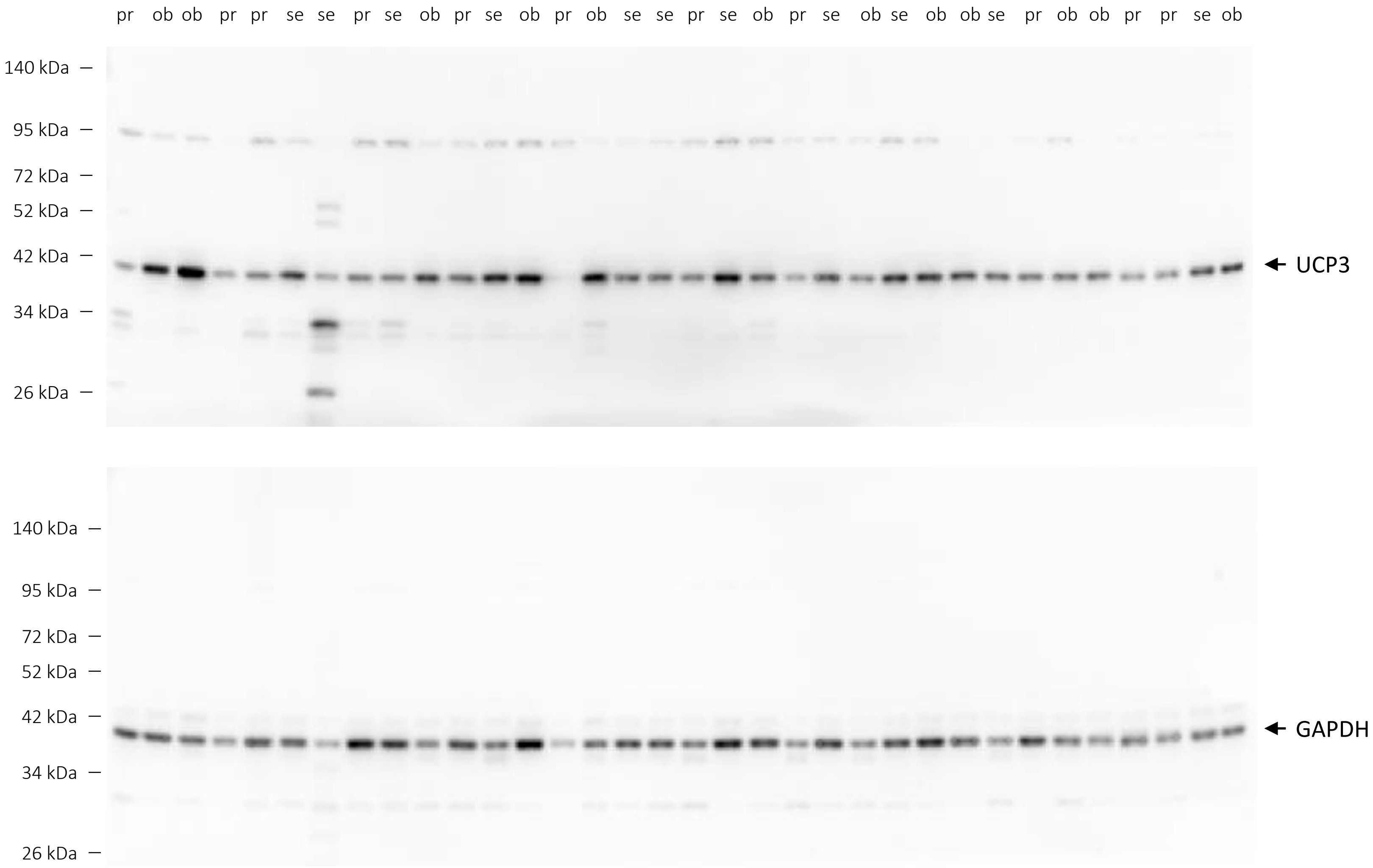

WB evaluation of MCU

Membrane was treated as followed

- Blocking 5% milk in TTBS
- anti-MCU (1:1000, O/N, 4°C)
- Anti-rabbit POD (1:10´000, 1h, RT)
- Developed
- Anti-GAPDH (1:5000, 1h, RT)
- Anti-mouse POD (1:5000, 1h, RT)
- developed

Ob = HFpEF untreated
Pr = HFpEF primary prevention
Se = HFpEF secondary prevention

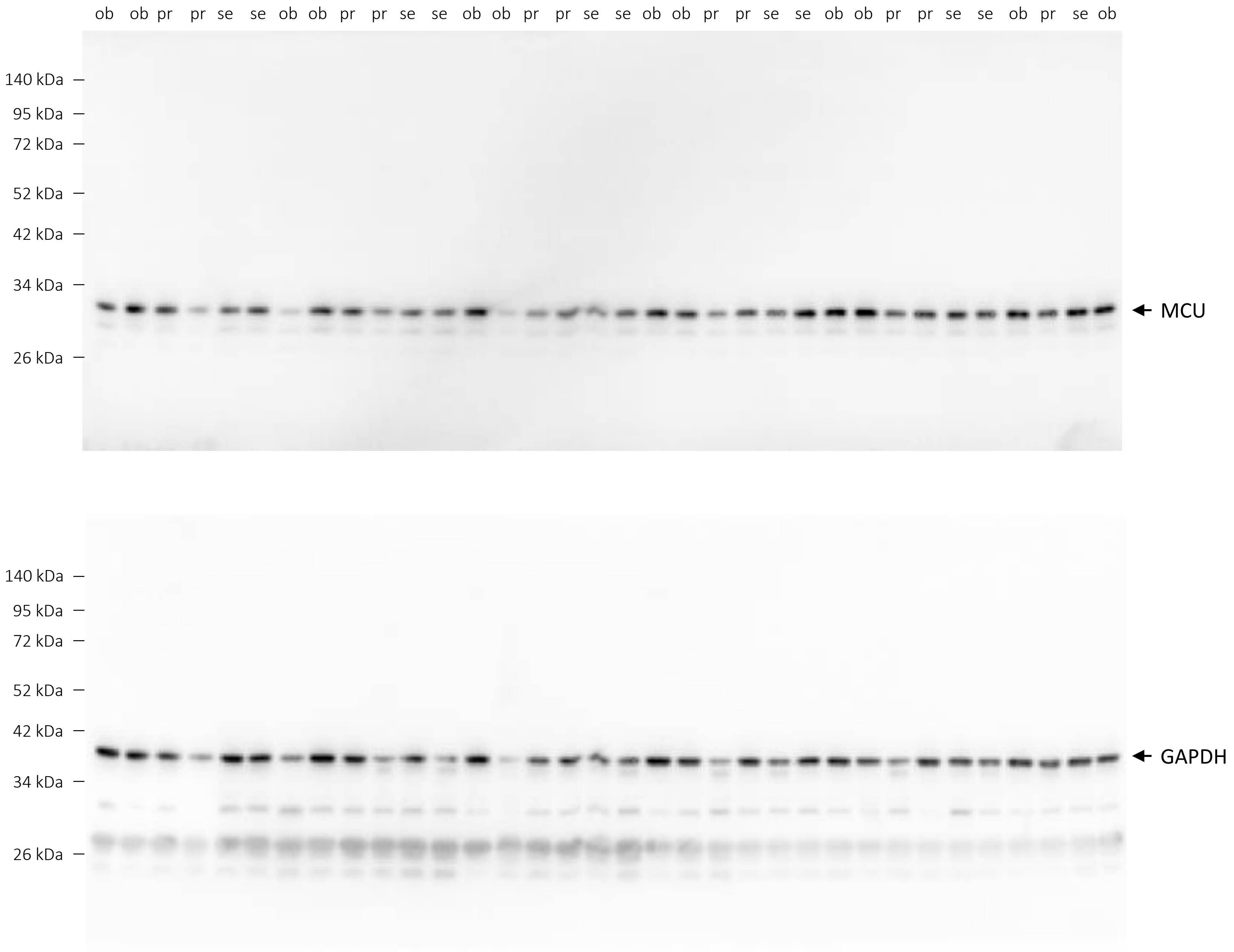

WB evaluation of MuRF1

Membrane was treated as followed

- Blocking 5% milk in TTBS
- anti-MuRF1 (1:200, O/N, 4°C)
- Anti-mouse POD (1:5000, 1h, RT)
- Developed
- Anti-GAPDH (1:5000, 1h, RT)
- Anti-mouse POD (1:5000, 1h, RT)
- developed

Ob = HFpEF untreated
Pr = HFpEF primary prevention
Se = HFpEF secondary prevention

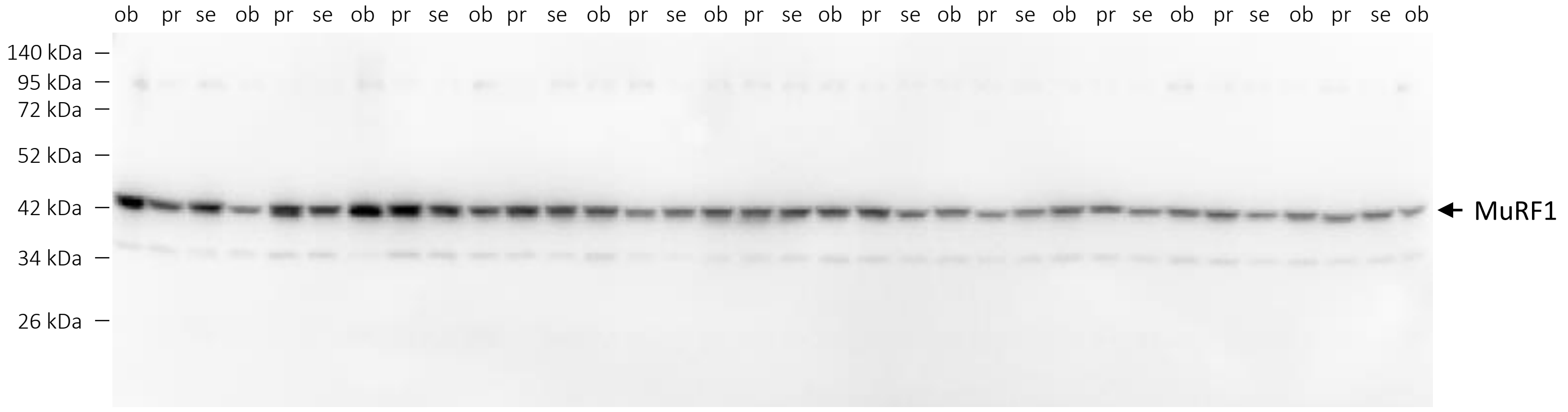

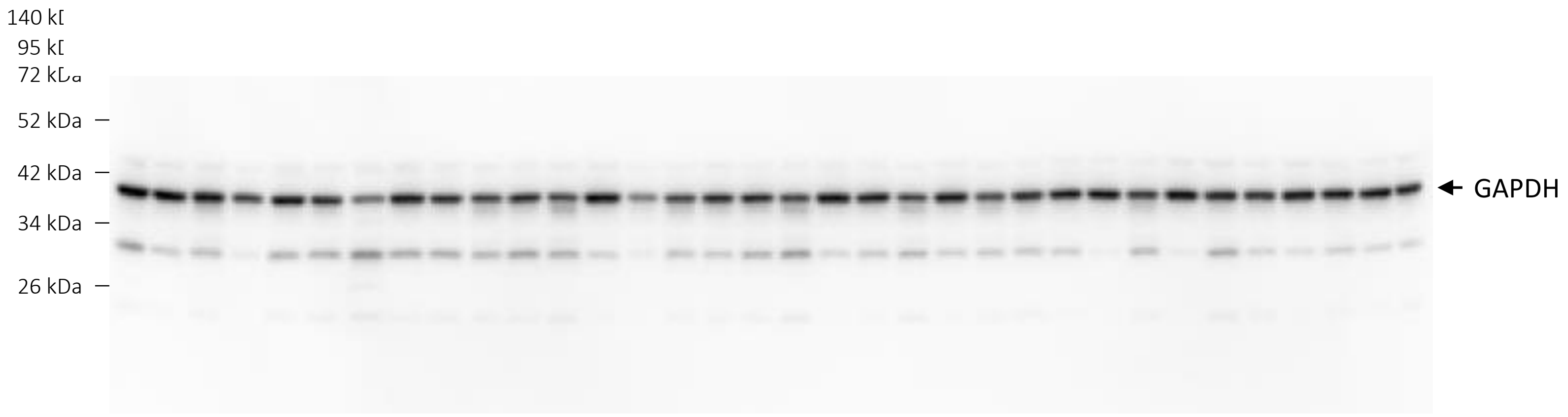

WB evaluation of MAFBx

Membrane was treated as followed

- Blocking 5% milk in TTBS
- anti-MAFBx (1:1000, O/N, 4°C)
- Anti-rabbit POD (1:10´000, 1h, RT)
- Developed
- Anti-GAPDH (1:5000, 1h, RT)
- Anti-mouse POD (1:5000, 1h, RT)
- developed

Ob = HFpEF untreated
Pr = HFpEF primary prevention
Se = HFpEF secondary prevention

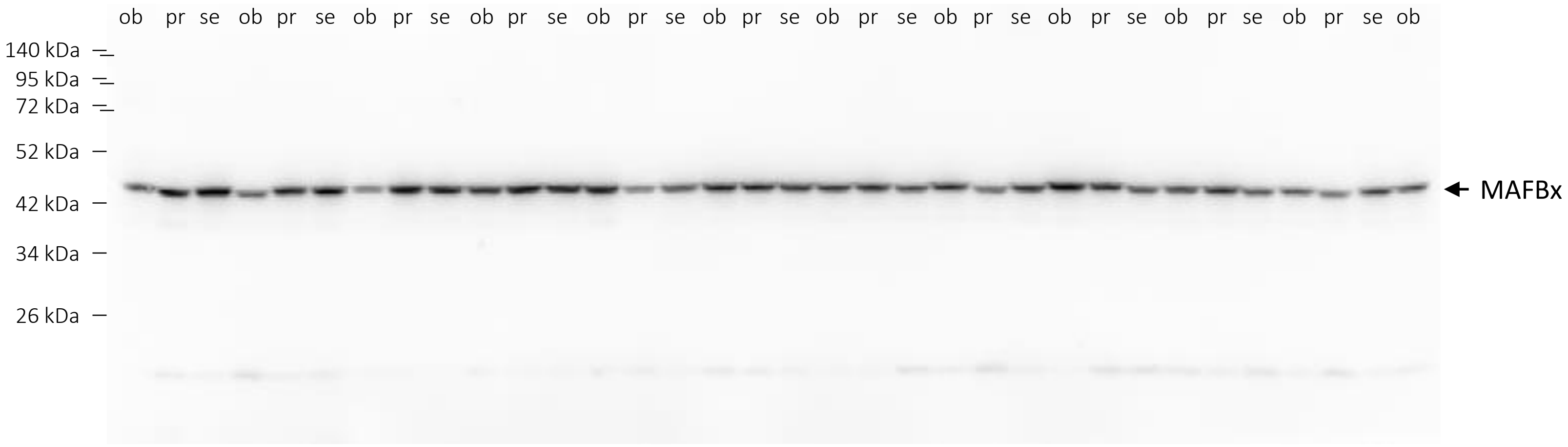

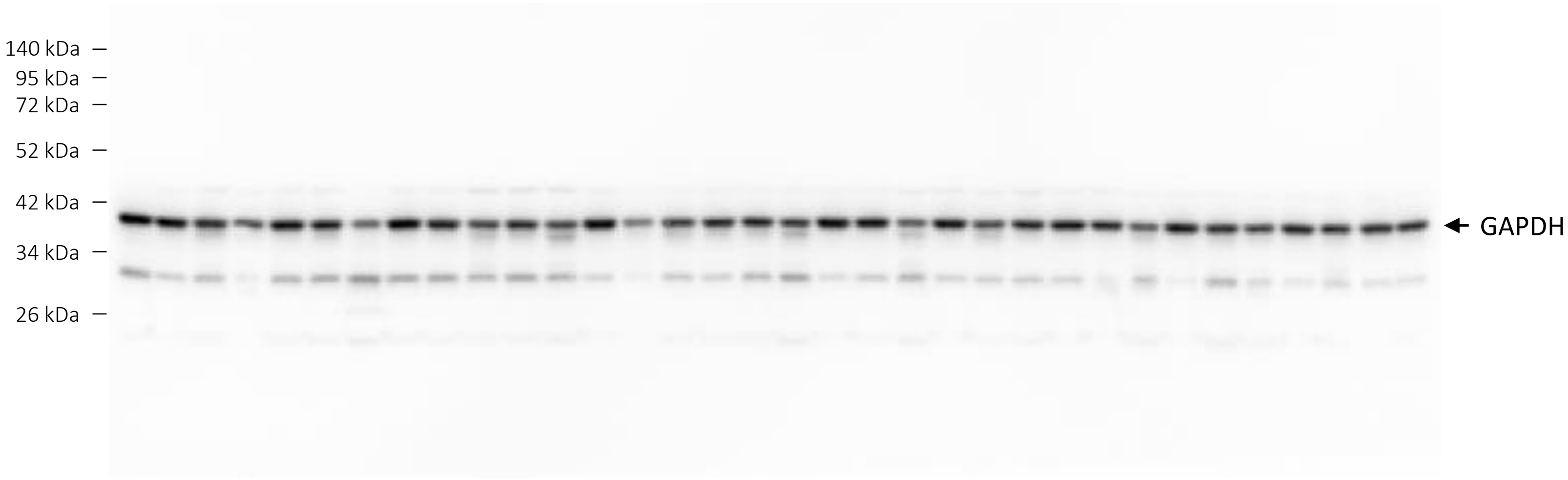

Supplement: Supplementary file 1 [file cells-13-00502-s001.zip › cells-2839120-supplementary.pdf]
